# Limiting Exciton Diffusion Enhances the Optical Response of CsPbBr_3_ Nanocrystal Films at High Excitation Densities

**DOI:** 10.1021/acs.nanolett.6c01064

**Published:** 2026-06-02

**Authors:** Simon P. S. Jessen, Jan Král, Eva Mihóková, Vojtěch Zabloudil, Michal Horák, Etiennette Auffray, Kateřina Děcká, Rosana M. Turtos

**Affiliations:** † Department of Physics and Astronomy, 1006 Aarhus University, 8000 Aarhus, Denmark; ‡ Faculty of Nuclear Sciences and Physical Engineering, 156923 Czech Technical University in Prague, 11519 Prague, Czech Republic; § Institute of Physics, 86889 Czech Academy of Sciences, 16200 Prague, Czech Republic; ∥ 30531 CERN, 1211 Meyrin, Switzerland; ⊥ Central European Institute of Technology, 48274 Brno University of Technology, 61200 Brno, Czech Republic

**Keywords:** lead halide perovskite nanocrystals, multiexciton dynamics, ultrafast spectroscopy, radiation detectors

## Abstract

Ultrafast radiation detection requires materials to maintain favorable optical properties under dense excitation. In this work, we investigate lead halide perovskite nanocrystal (NC) films using the Z -scan luminescence method and show that their optical response under dense excitation is strongly influenced by the choice of surface ligand. We specifically report that using long organic ligands has a positive effect on the optical performance compared to short inorganic ligands because it limits exciton diffusion between neighboring NCs. These conclusions are based on Z -scan luminescence data, a numerical model incorporating inter-NC exciton diffusion, and measurements of a heterostructured detector under ionizing radiation. In addition, encapsulating the NCs in a SiO_2_ shell is identified as an effective strategy to reduce inter-NC exciton diffusion while providing improved chemical stability. Together, the experimental results and numerical modeling presented in this work establish predictive tools for the design of nanocomposite materials with optimized ultrafast luminescence properties.

## Graphical abstract

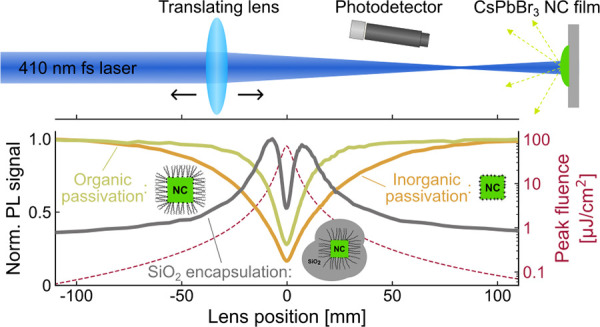

Cesium lead halide nanocrystals (NCs) have attracted significant attention from the scientific community due to their excellent optoelectronic properties. In past years, their narrow and spectrally tunable excitonic emission has become of interest for radiation detection.^1–4^ In particular, their ultrafast response under X-ray and γ-ray excitation originating from multiexcitonic recombination appears to be promising for fast-timing applications such as time-of-flight positron emission tomography (TOF PET).^5,6^ A recently proposed concept, chromatic calorimetry, represents a novel solution for high-energy physics that takes advantage of the narrow emission peak of lead halide perovskite (LHP) NCs to construct a segmented detector capable of performing longitudinal tomography of the shower profile.^2,7,8^

Despite high Z_eff_ atoms, NCs have a low stopping power for primary high-energy radiation due to their small volume. Consequently, applications of NCs in these concepts are envisaged in combination with high-density absorbers (scintillators themselves or not) capable of stopping the incoming primary high-energy radiation.^9^ The nanoscintillator itself is then excited by the generated secondary radiation, which usually exhibits large values of the energy loss per unit length. A notable example of this approach is the heterostructure concept proposed for TOF PET, in which layers of a high-density bulk scintillator alternate with layers of fast-emitting nanoscintillators, each providing part of the functionality.^10,11^

Due to the poor long-term chemical and environmental stability of the material, the NC layers in the detector design must be protected. Here, several options are available. NCs can be embedded in a protective polymer or glass matrix, forming a nanocomposite scintillator.^3,12,13^ Alternatively, NCs can be deposited on a (scintillating) substrate to form a thin film, which can then be encapsulated by depositing a protective overlayer, effectively isolating the NCs from the environment.^14,15^ Another approach is direct encapsulation of individual NCs within a protective shell. Among these, SiO_2_ encapsulation via tetraalkoxysilane (TEOS) hydrolysis is the most common, providing a simple and effective means of improving NC stability.^16–19^ Among these options, protected thin films are particularly attractive because densely packed NCs were shown to outperform isolated NCs under X-ray excitation due to more efficient conversion of the secondary electron shower generated by ionizing radiation.^20,21^ Understanding the optical properties of thin films under high-density excitation induced by high-energy radiation is therefore crucial for their application as radiation detectors.

In this work, we investigate the optical properties of drop-cast films of CsPbBr_3_ NCs from the same syntheses with different surface passivation techniques. To this end, we investigate the films using the interband Z -scan luminescence method at excitation densities between 10^15^ and 10^19^ cm^–3^. The core principle of this method is to examine the photoluminescence (PL) yield of a sample under varying excitation densities while maintaining a constant optical excitation magnitude, in contrast to conventional PL measurements, where excitation is varied via laser power. This is realized by focusing optical pulses at above-band-gap photon energies with a translating lens, as sketched in Figure 1a, while measuring a constant fraction of the emitted PL signal. Any deviation from a uniform response immediately reveals the presence of a nonlinear process in the sample, typically nonlinear quenching (NLQ). Examples of such Z scans are shown in Figure 1b, and they can be used to gain two major insights. First, this allows for an intricate identification and characterization of the physical processes responsible for luminescence and quenching in the sample, as demonstrated by numerous previous studies of various scintillators.^22–25^ Second, by probing the optical response of a sample in an excitation density regime relevant for the deposition of ionizing radiation, it is possible to effectively evaluate the efficiency of a nanoscintillator without the direct use of ionizing radiation. This is particularly valuable because current studies of nanoscintillators are generally hampered by the fact that they cannot be studied independently from their surrounding matrix, since a single NC cannot contain the energy of a γ photon.^26^ By the replication of conditions similar to those of γ-ray deposition through optical excitation, the problem imposed by a lack of total energy containment is completely avoided.

**Figure 1:**
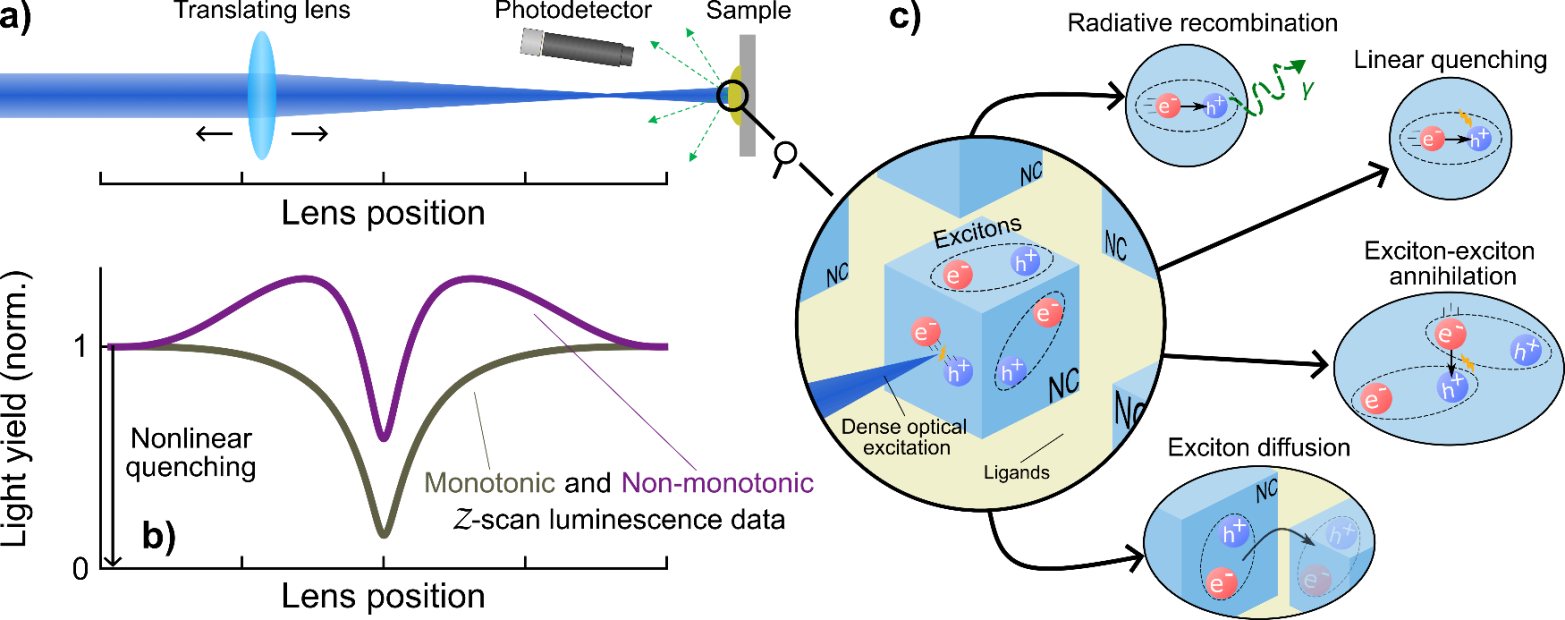
(a) Sketch of the Z -scan setup. (b) Examples of Z -scan luminescence data sets, illustrating the difference between a monotonic and a nonmonotonic PL response to increasing excitation density. (c) Schematic overview of the physical processes occurring in densely excited NCs considered in this study.

When a LHP NC is excited by either above-band-gap optical excitation or ionizing radiation, excitons are formed on an ultrafast (subpicosecond) time scale.^27^ Following spatially dense excitation, as is the case for ionizing radiation, multiple physical processes may occur, as illustrated in Figure 1c. A recent study using similar experimental methods demonstrated that the diffusion of excitons between NCs plays a crucial role in determining the optical response of drop-cast films of NCs at dense optical excitation.^25^ Because exciton–exciton annihilation (or bimolecular Auger recombination) is generally orders of magnitude faster than linear excitonic decay,^28^ inter-NC exciton diffusion can induce significant NLQ at excitation densities well below one exciton per NC. In this work, we investigate routes for enhancing the optical performance by controlling exciton diffusion.

The CsPbBr_3_ NCs under study were synthesized via the hot-injection method and subsequently modified to yield variants of surface passivation. The details of this procedure can be found in Sections S1 and S2. The three variants with surface passivation enabled by oleic acid (OA)/oleylamine (OAm), didodecyldimethylammonium bromide (DDAB), and ammonium hexafluorosilicate (AHFS) are shown in parts a–c of Figure 2, respectively, with the varying ligand lengths highlighted and actual interparticle distances determined by high-resolution transmission electron microscopy (TEM). While spacing correlates with the ligand length for the long-chain hydrocarbon ligands, surprisingly, the AHFS-treated sample exhibited the largest spacing, likely due to ligand interactions. OA/OAm and DDAB differ primarily in their passivation efficiency, with OA/OAm showing dynamic bonding with lower stability and DDAB providing improved surface coverage and stability. This translates to measured photoluminescence quantum yields (PLQYs) in solution of our NCs, with the OA/OAm ligand pair reaching only 22% and the DDAB modification achieving 67%. PLQY measurements of the AHFS-treated NCs confirm the excellent passivation properties of halide anions, with values exceeding 80%. Importantly, other than differences in their PLQY values, no substantial changes to the emission and absorption properties between these variants were observed, as shown in Figure 2d. Further details on the characterization of the NCs can be found in Section S3. The dense films of NCs for Z -scan measurements were then fabricated by drop-casting the solution onto a silicon substrate, as seen in Figure 2d. Unfortunately, films drop-casted from the OA/OAm-passivated samples were too fragile to be used for Z -scan measurements; for the discussion, see Section S4.

**Figure 2:**
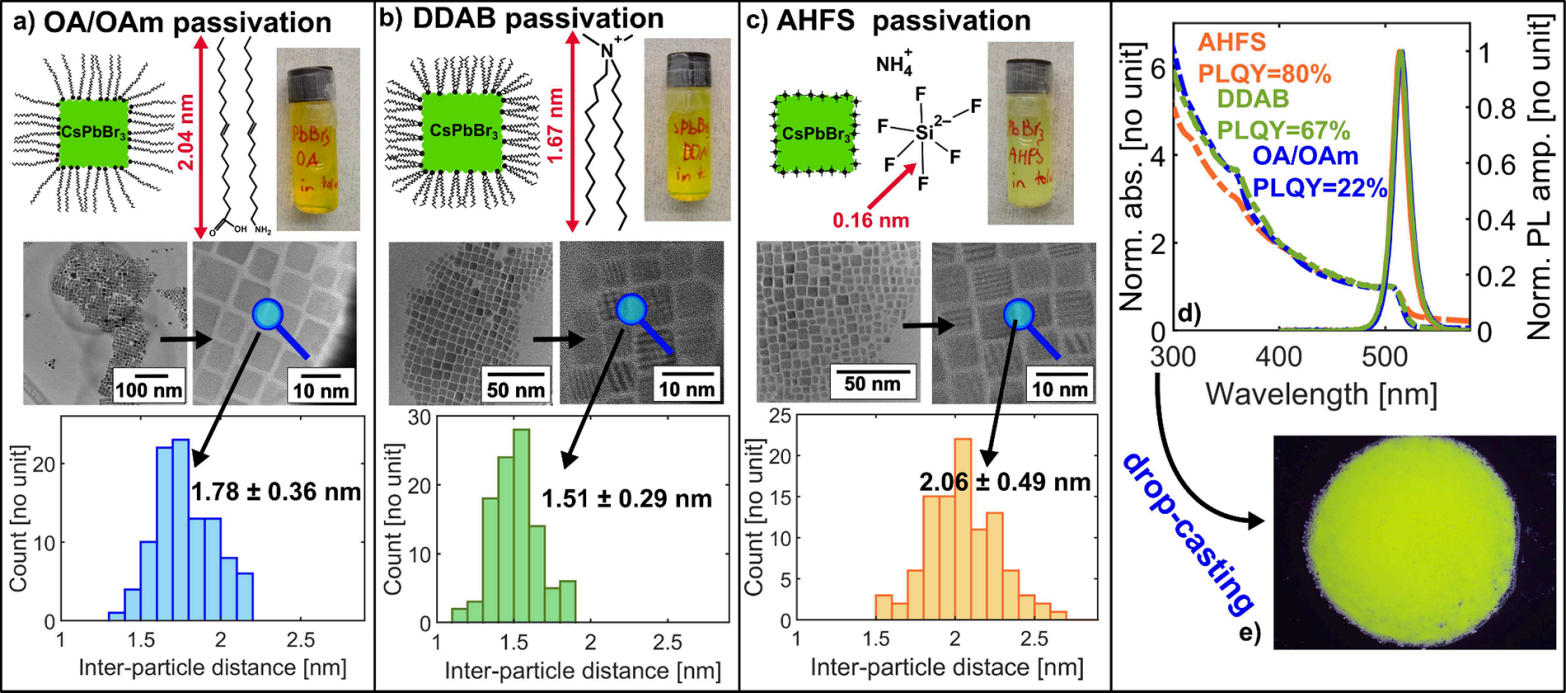
(a) Top: Schematic depiction of OA/OAm ligand pair modification with a structural formula and photograph of the sample. Bottom: TEM micrographs of the CsPbBr_3_ OA/OAm NCs and histograms of their interparticle distances. (b) Same as part a for DDAB modification. (c) Same as parts a and b for AHFS modification. (d) Normalized absorption and PL emission spectra of NCs from parts a–c. (e) Drop-casted layer of CsPbBr_3_ NCs prepared for Z -scan measurements.

Data from Z -scan luminescence measurements of drop-casted films of DDAB- and AHFS-passivated CsPbBr_3_ NCs are shown in Figure 3a. The measured PL signal at each lens position is plotted against the calculated peak excitation density in terms of excitons per unit volume in the sample. Such data sets are hereafter referred to as NLQ traces. The calculation of the peak excitation density assumes linear absorption and is based on an estimate of the absorption coefficient of the sample and careful beam characterization, the details of which can be found in Sections S4 and S5, respectively. The *x* axis of a NLQ trace (eq S3) can be converted to the peak number of excitons per NC, denoted as *n*_0,max_, by multiplying by the NC volume. For clarity, the raw Z -scan data, along with the measured peak fluence (eq S4), are shown as an inset in Figure 3a. Note that, because a NLQ trace is always normalized, it contains no information about the linear PLQY of a sample.

**Figure 3:**
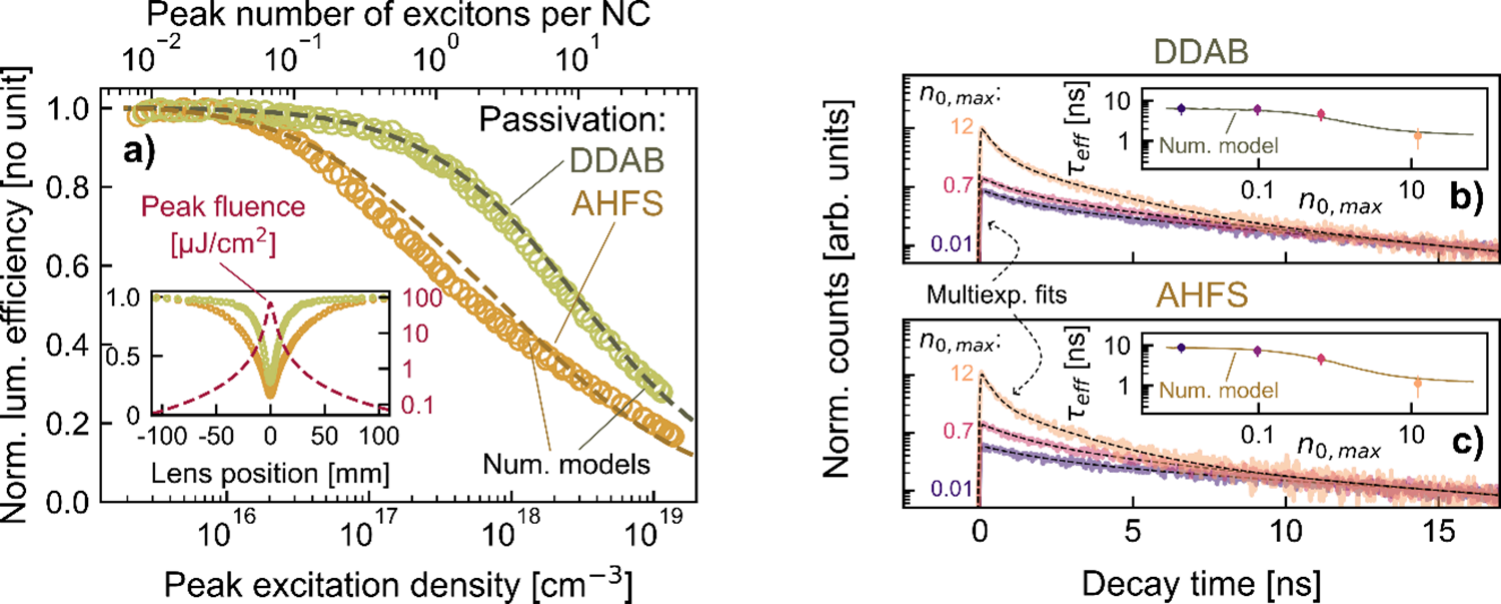
(a) NLQ traces of drop-casted samples of DDAB- and AHFS-passivated NCs (green and orange circles, respectively). Dotted lines represent predictions from the numerical model with different diffusion rates. The raw Z -scan data are presented as an inset. (b and c) Decay curves of DDAB- and AHFS-passivated samples at three peak numbers of excitons per NC, *n*_0,max_. Quadruple-exponential fits are included as black dotted lines. Amplitude-weighted effective decay times, τ_eff_, extracted from the measured (circles) and simulated (solid lines) decay curves are plotted in the insets on a log–log scale against *n*_0,max_. Each decay curve has been normalized at 15 ns.

We model the exciton dynamics in the drop-casted films of CsPbBr_3_ NCs following intense optical excitation using an entirely numerical approach. The model is similar to the one developed by Fratelli et al. in ref 20 and is described in detail in Section S6. Crucially, our model includes exciton diffusion, modeled as an effective Förster-type hopping process between neighboring NCs in the film, which was not considered in the previous work. Using this model, we can predict the normalized luminescence efficiency at a given peak number of excitons per NC with the following model parameters: linear luminescence efficiency, η; exciton lifetime, τ_X_; biexciton lifetime, τ_XX_; exciton diffusion rate, *R*_D_.

Upon examination of the NLQ traces of the samples passivated by DDAB and AHFS in Figure 3a, the sample with the shorter ligand shows stronger NLQ effects. This is evident from the fact that the PL signal of the AHFS-passivated sample begins to decrease due to NLQ at excitation densities well below 1 exciton/NC. This indicates that a considerable rate of exciton diffusion occurs in the sample. In contrast, NLQ only begins to significantly affect the PL signal for the DDAB-passivated sample at excitation densities around 1 exciton/NC, which indicates limited levels of exciton diffusion.

The difference between the NLQ traces of DDAB- and AHFS-passivated samples can be captured numerically by assigning different rates of exciton diffusion to the two samples. Simulated NLQ traces using the numerical model described in Section S6 are shown in Figure 3a. The linear decay rate, 1/τ_X_, is chosen as the inverse of the measured effective decay time at low fluence for the sample, while the biexciton lifetime, τ_XX_, is set to 350 ps (see Section S6). The diffusion rate, *R*_D_, is found by matching the simulated NLQ traces to the experimental data. For the DDAB- and AHFS-passivated samples, the value for *R*_D_ was set to 0.3 and 10 ns^–1^, respectively. A summary of all parameters used in the numerical model to reproduce the NLQ traces is provided in Table 1.

**Table 1:** Summary of the Key Characteristics of the Four Different Samples Shown in Figure 3a,c, Together with the Parameters Used in the Simulation of Their NLQ Traces

sample characteristics		model parameters			
surface passivation	PLQY in solution [%]	τ_X_ [ns]	τ_XX_ [ps]	*R*_D_ [ns^–1^]	η [%]
DDAB	62	6.4	350	0.3	62
AHFS	80	8.8	350	10	80
OA/OAm+SiO_2_	22	14.5	350	0	1.4
AHFS+SiO_2_	84	10.8	350	0	82

The numerical model can reproduce the measured NLQ traces with good accuracy. Any deviation between the model and the data is most likely caused by the simplicity of the numerical model. The model treats the drop-casted samples as an infinitely repeating crystal-like cubic structure of NCs, which is obviously not an accurate representation of the complicated microscopic sample structure. Nevertheless, the model supports the hypothesis that exciton diffusion is the primary cause of the difference between the NLQ traces of the samples with the two different surface passivation techniques.

The diffusion of excitons is modeled as Förster-type resonant energy transfer between neighboring NCs, with a transfer rate that depends not only on the inter-NC distance but also on factors such as PLQY, energetic disorder, and the dielectric environment around the NCs.^29^ The modeled exciton diffusion rates for the two samples are consistent with this framework and with previous studies showing that exciton transport in NC solids is not solely determined by inter-NC spacing but is strongly influenced by surface chemistry.^30,31^ In previous work, organic ligand shells have been shown to limit exciton diffusion by electronically isolating individual NCs.^32^ In contrast, inorganic or atomic surface passivation has been demonstrated to enhance electronic transport in NC solids, which is a common indicator of improved inter-NC coupling and reduced energetic disorder.^33^ Consistent with this picture, the higher PLQY of the AHFS-treated sample in solution suggests a more effective surface trap passivation, a factor that has previously been correlated with enhanced exciton mobility in NC films.^31^ Notably, the AHFS-treated sample exhibits a higher effective diffusion rate despite a larger average interparticle distance, indicating that inter-NC spacing alone cannot account for the observed trends. Regardless of the chemical origin of the different diffusion rates, the observed NLQ traces and numerical model highlight the negative impact of exciton diffusion on the overall luminescence efficiency of tightly packed samples of NCs at high excitation densities. Notably, controlling exciton diffusion independently of inter-NC spacing is particularly significant for applications requiring high stopping power, such as ionizing radiation detection.

Decay curves measured for the films drop-casted from the DDAB- and AHFS-passivated samples at selected excitation densities are shown in Figure 3b,c, respectively, along with multiexponential fits. Details on the experimental procedure and the multiexponential fitting can be found in Section S7. From the multiexponential fits, amplitude-weighted effective decay times are calculated (eq S12) and shown in the insets of Figure 3b,c. The measured decay curves can be compared to the numerical model by employing the same analysis (see Section S7) on the decay curves predicted by the model. The excitation-density-dependent effective decay times predicted by the numerical model are shown in the insets of Figure 3b,c. The numerical model captures the trend of a faster PL decay at higher excitation densities, which demonstrates that the essential features of the decay kinetics are captured by the model.

To validate the results obtained in Z -scan measurements, we tested the performance of the DDAB- and AHFS-passivated NCs under high excitation densities produced by ionizing radiation. To do so, we utilized the basics of the heterostructure concept of combining the high-stopping-power scintillator and NC layers and an experimental setup for coincidence time resolution measurement.^34^ The heterostructure consists of a 200-μm-thick layer of a dense scintillator Bi_4_Ge_3_O_12_ (BGO), a drop-casted film of NCs, and a layer of polystyrene (PS), as sketched in Figure 4a. Photoemission resulting from exposure to 511 ke V γ-radiation is measured with a silicon photomultiplier (SiPM). Further details on the preparation of the heterostructure and the experimental setup can be found in ref 5 and Section S8.

**Figure 4:**
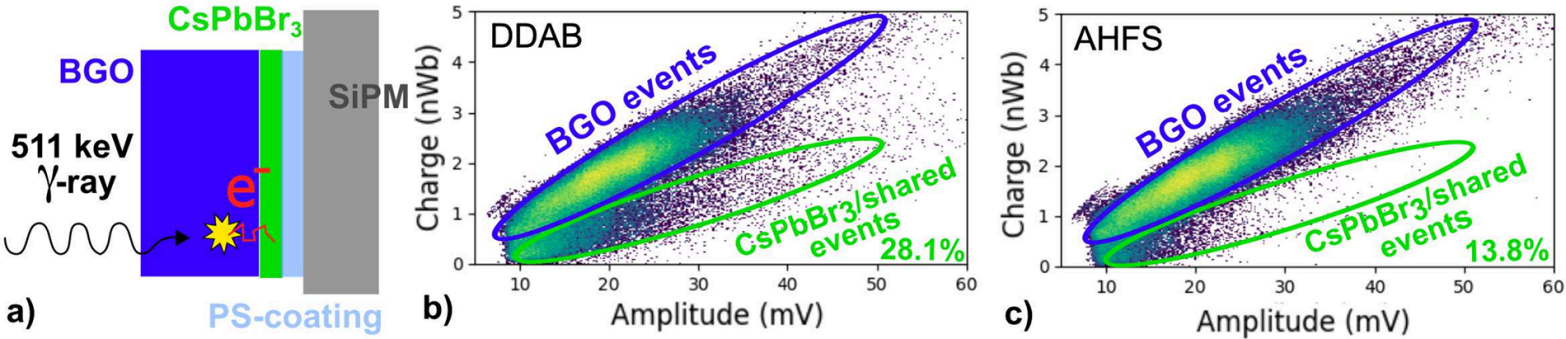
(a) Schematic depiction of the heterostructure exposed to γ-ray irradiation. (b and c) 2D charge-amplitude histograms using layers of either DDAB- or AHFS-treated NCs on a BGO scintillator. Regions corresponding to interactions purely in the BGO crystal and shared events are highlighted. Both histograms are normalized to the same total number of events.

When a 511 ke V γ-photon interacts with the heterostructure, it can either (1) deposit all of its energy in the dense bulk scintillator, (2) first interact with the BGO plate generating a photoelectron, which can subsequently travel and excite the NC layer, or (3) interact, although less likely, only with the NC layer. Thanks to the different scintillation mechanisms and kinetics of NCs and BGO, these events can be distinguished by pulse-shape discrimination in 2D charge-amplitude histograms, which are shown for the DDAB- and AHFS-treated samples in Figure 4b,c, respectively. Here, two distinct regions can be identified, a major one corresponding to the events in the BGO crystal and a second one with events of lower charge but relatively high amplitude corresponding to energy sharing or pure NC layer events. Looking at the number of events in this second region, one can compare the performance/light output of the NC layer with different ligands under mostly high-density excitation by shared secondary electrons. A clear difference can be observed between the samples with a DDAB-treated sample having 28.1% of all events in the second region in contrast to only 13.8% events for a AHFS-treated sample. These results correspond very well with performed Z -scan measurements showing significant quenching of the light emission from AHFS-treated NCs. Further discussion on the event discrimination is presented in Section S8.

Another strategy for limiting exciton diffusion in a tightly packed geometry such as a drop-casted film is to encapsulate each NC in a protective layer of SiO_2_. This technique also improves the long-term stability of the NCs.^19^ Two of the three NC modifications, notably with varying PLQY values, namely, the OA/OAm ligand pair and the AHFS treatment, were encapsulated in SiO_2_. The process of preparing the SiO_2_ particles with embedded CsPbBr_3_ NCs is described in detail in Section S2 and is schematically depicted in Figure 5a,b. For the TEM micrographs of the resulting structures, refer to ref 35. During the process, the PLQYs of the precursor NCs were preserved after encapsulation alongside their emission peaks, which are documented in Figure 5a,b. Only a minor red shift of the OA/OAm emission peak was observed, likely due to the degrading influence of ethanol released during the hydrolysis of TEOS and the labile surface coverage of the OA/OAm ligand pair.

**Figure 5:**
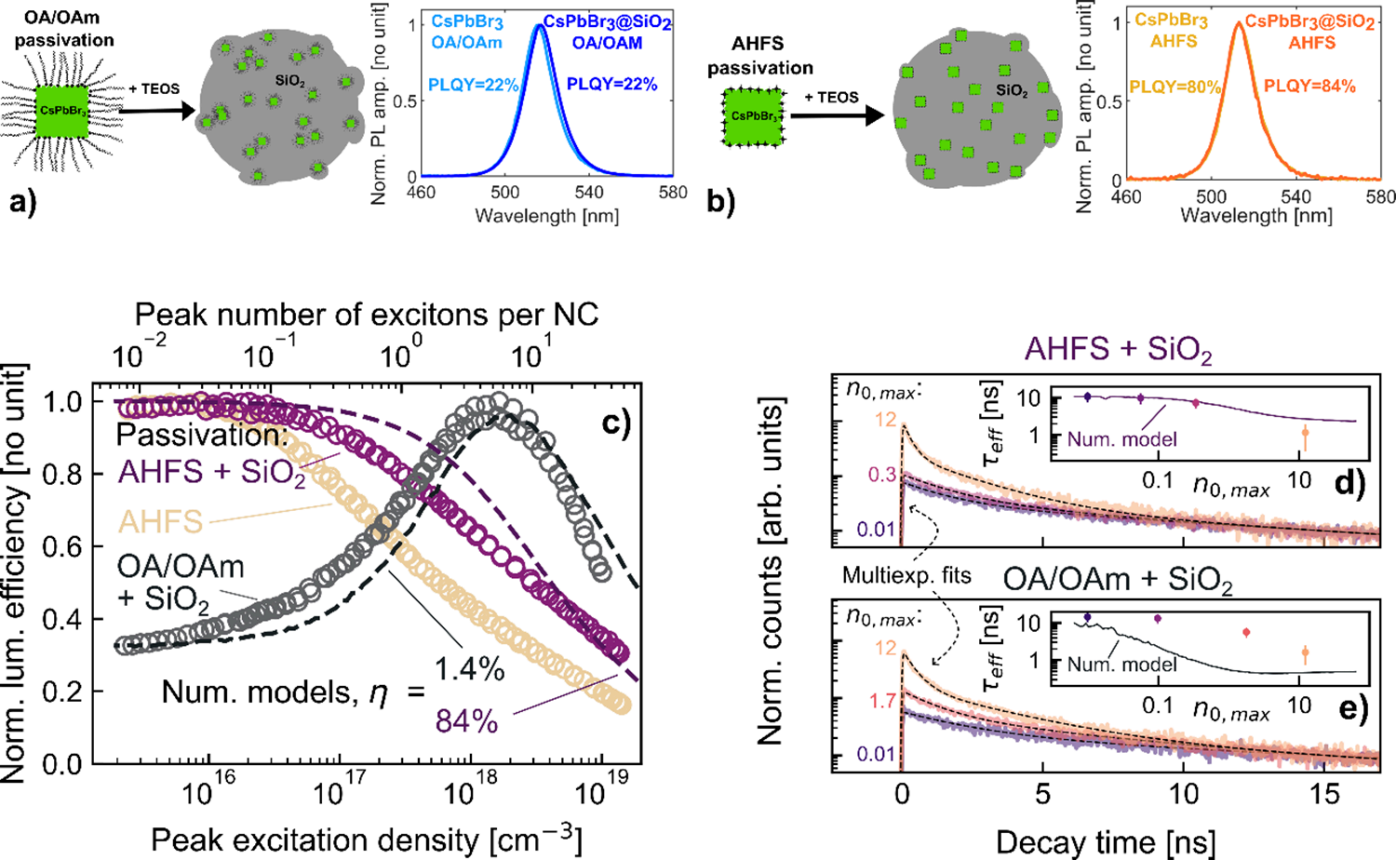
(a) Schematic depiction of the SiO_2_ encapsulation of OA/OAm-treated CsPbBr_3_ NCs with the respective PL emission spectra before and after encapsulation. (b) Same as part a for AHFS-treated NCs. (c) NLQ traces of the drop-casted samples of AHFS+SiO_2_- and OA/OAm+SiO_2_-passivated NCs (purple and gray circles, respectively). Dotted lines represent predictions from the numerical model with different linear PL efficiencies. For comparison, the measured NLQ trace of the non-SiO_2_-encapsulated AHFS-passivated sample is also included as golden circles. (d and e) Decay curves of the two SiO_2_-encapsulated samples at three excitation densities, along with multiexponential fits (black dotted lines). Effective decay times from the measured data (dots) and numerical model predictions (solid line) are shown in the insets.

NLQ traces of these two variants of SiO_2_-encapsulated CsPbBr_3_ NCs are shown in Figure 5c. For comparison, the NLQ trace of the non-SiO_2_-encapsulated AHFS-passivated sample is also shown in Figure 5c. Despite their very similar chemical compositions, the two SiO_2_-encapsulated samples exhibit markedly different NLQ traces. The NLQ trace of the AHFS+SiO_2_-passivated sample (with a high PLQY) is quite similar to that of the non-SiO_2_-encapsulated CsPbBr_3_ NCs, whereas the OA/OAm+SiO_2_-passivated sample (with a low PLQY) has a distinctly nonmonotonic shape. Note that the normalization of a nonmonotonic NLQ trace is ambiguous because one may normalize either to the PL signal at low excitation density or to the maximum PL signal, as they no longer coincide. In Figure 5c, the NLQ traces are normalized to the maximum PL signal for visual purposes.

It is clear from the NLQ traces presented in Figure 5c that SiO_2_ encapsulation has improved the performance of the AHFS-passivated sample. From the analysis presented earlier, we attribute this to lower levels of exciton diffusion. We model the NLQ traces of the two SiO_2_-encapsulated samples with the same numerical model used for the non-SiO_2_-encapsulated samples, as described in Section S6. In these samples, exciton diffusion is not considered, that is, *R*_D_ = 0, due to the large SiO_2_ shells separating the NCs. Using the measured effective decay time at low excitation density as τ_X_, assuming τ_XX_ = 350 ps, and manually selecting the linear PL efficiency η, the NLQ traces can be qualitatively reproduced, as shown in Figure 5c.

There appears to be a slightly larger discrepancy between the measured and simulated NLQ traces compared with samples consisting of non-SiO_2_-encapsulated NCs (Figure 3a). This is most likely due to the large structural disorder in the SiO_2_-encapsulated samples in comparison to the perfect lattice used in the simulations. We also note that the small red shift observed in the OA/OAm+SiO_2_ sample may indicate subtle changes in the NC size, aggregation, or defect landscape, which could also contribute to the observed discrepancies and its nonmonotonic behavior. The numerical model, however, still qualitatively captures the difference between the NLQ traces of the two samples. Crucially, the modeled values of η qualitatively agree with the QY measurements of the two samples in solution. That is, the model predicts a lower linear PL efficiency for the low-QY sample; however, the predicted value of η = 1.4% does not quantitatively match the PLQY measured for the OA/OAm+SiO_2_ sample in solution. This discrepancy reflects limitations in the current numerical model, and η should therefore be regarded as an effective fitting parameter rather than a direct measure of the intrinsic PLQY. Even so, the measured and simulated NLQ traces are consistent with a link between a nonmonotonic NLQ trace and a low linear PL efficiency. Because nonmonotonic NLQ responses are of fundamental interest for unraveling carrier dynamics, this point will be discussed in more detail later.

Decay curves of the SiO_2_-encapsulated samples at selected excitation densities are shown in Figure 5b,c for the AHFS+SiO_2_ and OA/OAm+SiO_2_ samples, respectively. The decay curves are fitted using multiexponential functions, from which an effective decay time is extracted. The measured effective decay times and the effective decay times predicted by the numerical model are shown in the insets of Figure 5b,c. As expected, the PL decay becomes faster at higher excitation densities, which is reproduced rather accurately by the numerical model for the AHFS+SiO_2_ sample. The same agreement is not observed for the OA/OAm+SiO_2_ sample because the numerical model predicts a much faster PL decay at high excitation densities than is experimentally observed. At present, we do not have a satisfactory explanation for this discrepancy.

While the optical response of the OA/OAm+SiO_2_-passivated sample cannot be directly compared to a non-SiO_2_-encapsulated counterpart, the observed nonmonotonic NLQ trace is nevertheless notable because such behavior is extremely rare. It has, to our knowledge, only been observed in bulk, single-crystal samples of ZnO,^23,36^ undoped or lightly doped CsI,^23,37,38^ and MAPbBr_3_.^39^ The explanation for this behavior varies between materials and is not completely understood in the literature. From a phenomenological perspective, a nonmonotonic response during interband Z -scan luminescence measurements is evidence of a radiative term with a higher-order dependence on the excitation density than the dominant quenching term. In LHP NCs where exciton formation can safely be considered ultrafast,^27^ NLQ is exclusively governed by exciton dynamics.^23^ This means that the sole explanation for a nonmonotonic behavior must include a higher-order excitonic radiative term, such as biexcitonic emission.

When simulating NLQ traces using different values of η, it became apparent that a low linear PL efficiency is a prerequisite for a nonmonotonic NLQ trace. This is illustrated in Figure 6, where simulations of five NLQ traces using parameters identical with those used to replicate the OA/OAm+SiO_2_ sample (Table 1) except for η, which is varied between 1% and 50%. These simulations clearly show how increasing the luminescence efficiency of the sample gradually erases the nonmonotonic “hump” in the NLQ trace until it is essentially monotonic for η ≥ 10%. In the model, this can be explained by considering that the PL signal has contributions from both linear and nonlinear channels. However, when the linear luminescence efficiency becomes too high, the contribution from biexcitonic emission is “drowned out” and the NLQ trace becomes monotonic. For visual purposes, the NLQ traces of the AHFS+SiO_2_ and OA/OAm+SiO_2_ samples are also shown in Figure 6, where all traces have been normalized to their value at low excitation density.

**Figure 6:**
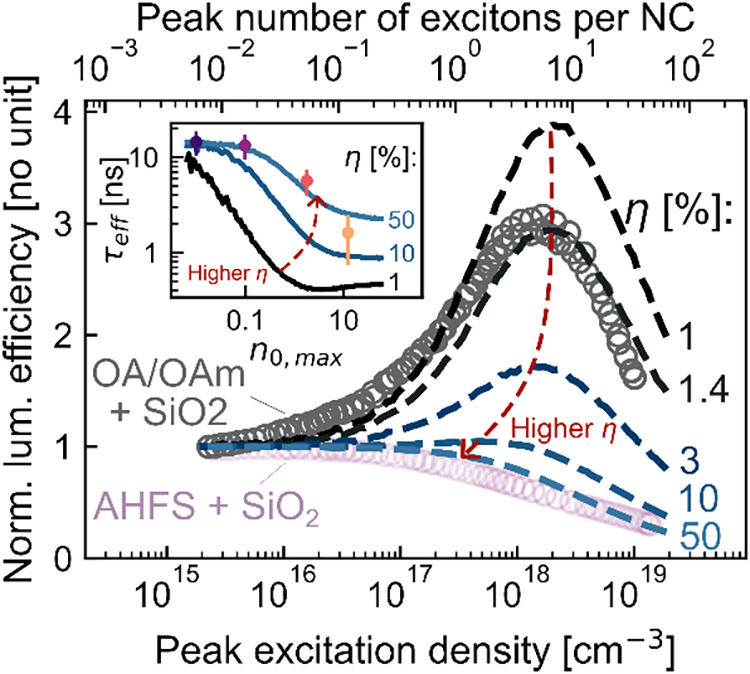
Graphical summary of the influence of the linear luminescence efficiency, η, on the simulated NLQ traces and decay curves. Simulated NLQ traces are shown as dotted lines for η values of 1%, 1.4%, 3%, 10%, and 50%, together with the measured NLQ traces of the two drop-casted samples of SiO_2_-encapsulated NCs. In the inset, the measured effective decay times of the OA/OAm+SiO_2_ sample (dots) are compared with simulated excitation-density-dependent effective decay times using the numerical model with three different η values (solid lines).

The simulations presented in Figure 6 can, in broad strokes, be compared to another physical system: CsI scintillating crystals. Whereas undoped CsI exhibits a nonmonotonic NLQ trace, gradually doping with low levels of Tl (greater than 0.1%) increases the light yield of the crystal^40^ and removes the nonmonotonicity.^37^ Because the origin of the nonmonotonic NLQ trace of CsI is not completely understood, as it is hypothesized to arise from either a competition between hot-electron trapping and exciton formation^37^ or radiation from a coupled state of two excitons,^38^ it is difficult to quantitatively compare the two systems. However, the studies presented here support the general observation that a nonmonotonic NLQ trace is associated with a low light yield.

The simulated influence of the linear PL efficiency on the effective decay time is shown in the inset of Figure 6, along with the measured effective decay times for the low-QY sample. These simulations indicate that a higher PL efficiency is associated with a slower decrease in decay times at increasing excitation densities. This is unsurprising because biexcitonic decay is faster than linear radiative recombination. Unfortunately, it seems that the measured effective decay times are better reproduced with a much higher value of η compared to that inferred from the NLQ trace. Further experimental and numerical studies of materials exhibiting nonmonotonic NLQ traces are therefore needed to fully understand their luminescence and quenching processes.

To summarize the finding presented in this work, we show that the surface passivation technique has a strong influence on the optical properties of drop-casted CsPbBr_3_ NCs thin films. We specifically conclude that suppressing inter-NC exciton diffusion improves the luminescence efficiency of the films under spatially dense excitation. We base this finding on Z -scan luminescence measurements and numerical modeling combined with measurements using ionizing radiation in a heterostructured detector setup. We also identify SiO_2_ encapsulation of the NCs as a viable strategy to limit exciton diffusion. Future studies should investigate whether this improvement also translates into a better performance when excited by ionizing radiation. The present work establishes a predictive pathway for the optimization and design of NC-based ionizing radiation detectors, showing that controlled optical excitation enables the bypassing of full detector construction while directly assessing and forecasting the material performance for next-generation particle detection.

## Supplementary Material



## Notes

List of used chemicals, nanocrystal synthesis and surface modification, nanocrystal characterization, absorption coefficients of the samples, Z -scan luminescence setup and beam characterization, numerical model, time-resolved measurements and multiexponential fitting, and measurements using 511 ke V excitation (PDF)
