# Supplementary material for: Limiting Exciton Diffusion Enhances the Optical Response of CsPbBr3 Nanocrystal Films at High Excitation Densities
Source: Nano Lett. 2026 Jun 2;26(23):7597–604. doi: 10.1021/acs.nanolett.6c01064 (PMC13281525; doi:10.1021/acs.nanolett.6c01064)
Supplement: Supplementary file 1 [file nl6c01064_si_001.pdf]

Supporting information: Limiting exciton diffusion enhances the optical response of $CsPbBr_3$ nanocrystal films at high excitation densities

Simon Jessen[1], Jan Král[2,3], Eva Mihóková[2,3], Vojtech Zabloudil[2,4], Michal Horák[5], Etiennette Auffray[4], Kateřina Děcká[2], and Rosana M. Turtos*[1]

[1]Department of Physics and Astronomy, Aarhus University, 8000 Aarhus, Denmark
[2]Department of Nuclear Chemistry, Czech Technical University in Prague, 115 19 Prague, Czech Republic
[3]Institute of Physics, Czech Academy of Sciences, 162 00 Prague, Czech Republic
[4]CERN, 1211 Meyrin, Switzerland
[5]Central European Institute of Technology, Brno University of Technology, 612 00 Brno, Czech Republic

*Email: ro.turtos@phys.au.dk

Contents

S1 Chemicals used 2

S2 Nanocrystal synthesis and surface modification 2

S3 Nanocrystal characterization 3

S4 Absorption coefficients of the samples 3

S5 $\mathcal{Z}$-scan luminescence setup and beam characterization 4

S6 Numerical model 4

S7 Time-resolved measurements and multiexponential fitting 6

S8 Measurements using 511 keV excitation 7

S1 Chemicals used

Ammonium hexafluorosilicate (AHFS, 98%, Thermo Scientific), Cs_2CO_3 (99.9%, Sigma-Aldrich), didodecyldimethylammonium bromide (DDAB, 98%, Sigma-Aldrich), ethyl acetate (p.a., PENTA), 1-octadecene (ODE, 90%, Sigma-Aldrich), oleic acid (OA, 90%, Sigma-Aldrich), oleylamine (OAm, 70%, Sigma-Aldrich), $PbBr_2$ (99.999%, Sigma-Aldrich), tetraethylorthosilicate (TEOS, ≥99.0% (GC), Sigma-Aldrich) and toluene (anhydrous, 99.8%, Sigma-Aldrich) were used. All chemicals were used as received without further purification unless otherwise stated.

S2 Nanocrystal synthesis and surface modification

$CsPbBr_3$ NCs were synthesized by the standard hot-injection method following a slightly modified version of the procedure published by Protesescu et al. in Ref. S1. First, 0.4 M cesium oleate octadecene solution was prepared A total of 1.303 g of cesium carbonate (4 mmol), 12.7 mL of oleic acid (40 mmol), and 7.3 mL of 1-octadecene was loaded into 100 mL three-necked flask and connected to a Schlenk line. A high 5:1 ratio of OA:Cs was used for solution to be soluble at room temperature.[S2] The reaction mixture was subjected to two cycles of evacuation under vigorous stirring and refilling with argon, and then was heated up under vacuum to 110 °C. After 1 h, all cesium carbonate was dissolved. Then, 0.752 mmoL of $PbBr_2$, 20 mL of ODE, 2 mL of oleylamine (OAm), and 1.78 mL of OA, were mixed in 100 mL 3-neck flask and degassed at 110 °C under vacuum for 1 h. After that, the mixture was heated up under argon atmosphere and 0.5 mL of pre-synthesized cesium oleate solution was injected at 170 °C.

Following the synthesis, various post-synthetic ligand exchanges and surface treatments were employed to yield nanocrystals with varying surface passivation producing films with different packing and photoluminescence quantum yields (PLQY). All these NC modifications for the experiments were obtained from one synthetic batch ensuring the same NC core and size dispersion for all samples.

OA and OAm passivated NCs – long ligand, low PLQY

The ice-quenched reaction mixture was centrifuged at 10 950 g for 5 minutes and the sediment was redispersed in toluene to obtain a final OA/OAm colloidal solution.

DDAB passivated NCs – long ligand, high PLQY

The ice-quenched reaction mixture was mixed with 55 mM DDAB toluene solution to perform the ligand exchange (volume ratio 3:2). After 2 minutes of vigorous stirring, the NCs were precipitated by ethyl acetate (15 mL per 3 mL of crude reaction mixture) and isolated by centrifugation at 10 950 g for 5 minutes. The final DDAB colloidal solution was obtained by redispersing the sediment in toluene, additional details about the ligand exchange can be found in Ref. S3.

AHFS passivated NCs – short ligand, high PLQY

To perform AHFS ligand exchange, OA/OAm passivated NCs in toluene were mixed with 2M aqueous solution of AHFS. The ratio of 0.6:1 mg of AHFS in water and $CsPbBr_3$ NCs in toluene was used. After 5 minutes of vigorous stirring, the treated NCs were separated by centrifugation at 18 235 g and redispersed again in toluene. Further details about the passivation can be found in Ref. S4.

SiO_2-encapsulated NCs

To encapsulate selected NCs in an SiO_2 shell, 20 µL tetraethoxysislane (TEOS) per mg of NCs was added to the NC colloidal solution in toluene. The solution was stirred and left to dry at 45 °C in a drying chamber overnight. The SiO_2-encapsulated NCs were then redispersed in toluene by ultrasonication. Using this procudure, OA/OAm and AHFS NCs were encapsulated yielding two variants with different PLQY values.

S3 Nanocrystal characterization

PL emission and transmittance spectra were obtained using a FluoroMax spectrofluorometer (Horiba Jobin Yvon). Synthesized nanocrystals were handled under air and all the measurements were performed at the laboratory temperature. Excitation wavelength for the PL emission measurements was 320 nm. The photoluminescence quantum yield measurements were performed by the FLUOROLOG-QM^{TM} modular research fluorometer (Horiba Scientific), equipped with 121 mm integrating sphere QuantaPhi-2 with Spectralon as reflective surface. The excitation light was provided by the steady-state Xe lamp, emission was registered by the PMT R13456 (Hamamatsu). High-resolution transmission electron microscopy (TEM) imaging was performed on the FEI Titan Themis 60-300 cubed (TITAN) microscope at 300 kV. The samples for the TEM analysis were fabricated by drop-casting 1 µL of the solution on a commercially available ultrathin carbon membrane for TEM (PELCO Ultrathin carbon film on lacey carbon support film on a copper 400 mesh). The inter-particle distances were evaluated on the basis of 100 individual measurements of inter-particle distance for each ligand modification. These measurements were taken at three different areas of the sample. The resulting interval, presented in Figure 2 of the main text, has been determined as the arithmetic average $\overline{d}$ plus-minus two times the standard deviation 2σ, thus ensuring the coverage of 95% of all measured values. The values were calculated as follows:

$$\overline{d} = \frac{1}{n}\sum_{i=1}^{n} d_i = \frac{1}{100}\sum_{i=1}^{100} d_i$$

$$2\sigma = 2 \cdot \sqrt{\frac{\sum_{i=1}^{n}\left(d_i - \overline{d}\right)^2}{n-1}} = 2 \cdot \sqrt{\frac{\sum_{i=1}^{n}\left(d_i - \overline{d}\right)^2}{99}}.$$

The instrumental uncertainty was neglected as the information limit of the aberration corrected transmission electron microscope is below 100 pm and so considerably smaller than calculated 2σ values.

S4 Absorption coefficients of the samples

The linear absorption coefficient, α, of the drop-cast films of NCs is estimated from transmission measurements of the NCs in solution. The measured absorption coefficients of the NCs at 410 nm excitation are $7.7 \times 10^4\,\mathrm{cm}^{-1}$ and $8.7 \times 10^4\,\mathrm{cm}^{-1}$ when suspended in hexane and toluene, respectively. Since the refractive index of toluene at 410 nm ($n = 1.52$[S5]) is similar to the refractive index of DDAB ($n = 1.526$ according to the CAS database, though no wavelength is specified[S6]), and the refractive index of hexane ($n = 1.39$[S5]) is similar to that of SiO_2 ($n = 1.47$[S7]), an absorption coefficient of $8.7 \times 10^4\,\mathrm{cm}^{-1}$ is used for the DDAB sample, while an absorption coefficient of $7.7 \times 10^4\,\mathrm{cm}^{-1}$ is used for the AHFS and SiO_2-encapsulated samples. The estimates are chosen in accordance with the Maxwell-Garnett effective medium approach, where the contribution from the medium enters through its refractive index.[S8]

As mentioned in the main text, the calculation of excitation density in the films requires that we excite them in the linear absorption regime. This is true as long as $\alpha \gg I_{\mathrm{peak}}\beta$, were I_{peak} is the peak intensity of the optical pulse and β is the two-photon absorption coefficient of the film. The optical pulse duration, τ, is around 100 fs and as shown in the inset of Fig. 3**a** in the main text, we keep the peak fluence, F_0, (see the following section Section S5) below $100\,\mu\mathrm{J\,cm}^{-2}$. Therefore, the maximum peak intensity is around

$$I_{\text{peak, max}} = \sqrt{\frac{4\ln 2}{\pi}}\frac{F_{0,\,\max}}{\tau} \approx 1\,\mathrm{GW\,cm}^{-2}. \tag{S1}$$

Reported values of β for drop-cast $CsPbBr_3$ NC films include $28.6\,\mathrm{cm\,GW}^{-1}$ at 800 nm.[S9] However, as β has been shown to increase with linear absorption,[S10] it may be significantly higher at 400 nm; indeed, a polycrystalline $CsPbBr_3$ film exhibits β up to $4 \times 10^3\,\mathrm{cm\,GW}^{-1}$ at 400 nm.[S11] Even using this conservative upper bound, $I_{\mathrm{peak}}\beta$ reaches at most 5% of the linear absorption coefficient in our peak fluence range, confirming that excitation remains in the linear absorption regime.

The $\mathcal{Z}$-scan measurement requires preparation of at least a µm thick layer to reassure full light absorption. To do so, repeated drop casting of $CsPbBr_3$ NC colloidal solution on the substrate was necessary. While

for AHFS and DDAB samples successful stacking of NCs layers on the substrate with additional drops was achieved, for OA/OAm ligand pair, we struggled to prepare a thick enough sample with most of the NCs concentrating at the edges of the substrate as the solvent evaporated. This different behavior of NCs with different ligands can be explained by varying affinities between the ligand and the solvent. Even though chemically similar, DDAB and OA/OAm differ in strength of passivation mostly due to varying interaction strength with solvent, for DDAB being much more repulsive. This might translate to NCs stacking during solvent evaporation, causing much better cohesion of DDAB-passivated NCs.[S12]

S5 $\mathcal{Z}$-scan luminescence setup and beam characterization

The $CsPbBr_3$ NCs are investigated using the interband $\mathcal{Z}$-scan luminescence method with a frequency-doubled Ti:sapphire fs-laser at a wavelength of 410 nm or photon energy of 3 eV. As sketched in Fig. 1**a** in the main text, the optical pulses are focused onto the sample by a lens mounted on a translating stage, which enables a variable fluence to be delivered to the sample while keeping the average optical energy constant. A detailed description of the method and setup can be found in Ref. S13. The PL signal from the sample is measured using an ET Enterprises 9107B photomultiplier tube (PMT) operating at 700 V.

Before performing a $\mathcal{Z}$-scan, the beam shape is carefully measured at multiple lens positions using a Thorlabs BP209-VIS/M scanning slit beam profiler. The measured beam profiles are described well by 2D Gaussian functions from which the beam widths in the sample plane, $w_{x,y}$, are determined. The beam widths at each lens position, $\mathcal{Z}$, are then fitted to,

$$w_{x,y}(\mathcal{Z}) = w_0\sqrt{1+\left(M^2\frac{\lambda\mathcal{Z}}{\pi w_0^2}\right)^2}, \tag{S2}$$

where $\mathcal{Z}=0$ is defined as the lens position that places the beam waist in the sample plane, w_0 is the beam waist radius, and M^2 is the unitless beam quality factor. Using a cylindrical coordinate system where z is parallel to the beam path with $z=0$ at the sample surface and assuming a linear absorption coefficient, α, the initial distribution of excitons created in the sample is given by

$$n(\vec{r},z;\mathcal{Z}) = n_{\text{peak}}(\mathcal{Z})\exp\left(\frac{-2r^2}{w_x(\mathcal{Z})w_y(\mathcal{Z})}-\alpha z\right). \tag{S3}$$

The peak initial excitation density, n_{peak}, is proportional to the peak laser fluence, F_0, at each lens position

$$n_{\text{peak}}(\mathcal{Z}) = (1-R)\frac{\alpha F_0(\mathcal{Z})}{h\nu}, \tag{S4}$$

where

$$F_0(\mathcal{Z}) = \frac{2E_p}{w_x(\mathcal{Z})w_y(\mathcal{Z})}, \tag{S5}$$

$E_p/h\nu$ is the number of photons in the optical pulse, and R is the reflection coefficient of the sample.

S6 Numerical model

We model the exciton dynamics in the drop-cast films of $CsPbBr_3$ NCs following intense optical excitation using an entirely numerical approach. The model is inspired by the one developed for isolated NCs by Fratelli et al. in Ref. S14. The dense film of NCs is modeled as a cubic grid with equal spacing, a, and the initial distribution of excitons is drawn from a distribution equal to Eq. (S3). When created, the population of excitons evolves as each exciton can undergo five different processes:

a) It can decay radiatively with a rate of R_X.

b) It can decay nonradiatively with a rate of Q_X.

c) If it inhabits a NC with more than one exciton, it can decay nonradiatively with a rate of $1/\tau_{XX}$ times the number of possible unique pairs of excitons in the NC.

d) If it inhabits a NC with exactly two excitons, it can decay radiatively with a rate of $4R_X$.

e) It can move to a neighboring NC with a rate of R_{D}.

Numerically, these processes are modeled by writing a rate equation for each populated NC and then propagating the system over discrete time steps. The rate equation for a NC with N excitons, at a given position of (x,y,z) in the grid, is

$$\frac{dN_{x,y,z}}{dt} = -(R_X + Q_X)N_{x,y,z} - \frac{1}{\tau_{XX}}\binom{N_{x,y,z}}{2} - 4R_X N_{x,y,z}\delta_{N_{x,y,z},2} \tag{S6}$$

$$- R_{\mathrm{d}}N_{x,y,z} + \frac{R_{\mathrm{d}}}{M}\sum_{(i,j,k)\in\{-1,0,1\}} w_{i,j,k}N_{x+i,y+j,z+k}, \tag{S7}$$

where the rate of movement to each neighbor is weighted according to the distance to the neighbors,

$$w_{i,j,k} = \begin{cases} 0, & \text{if } |i|+|j|+|k| = 0 \\ (|i|+|j|+|k|)^{-3}, & \text{otherwise} \end{cases}. \tag{S8}$$

The total rate of movement is normalized to be equal to R_d for all NCs,

$$M = \sum_{(i,j,k)\in\{-1,0,1\}} w_{i,j,k} = 6 + \frac{3}{2} + \frac{8}{27} = \frac{421}{54}. \tag{S9}$$

The weighting factors, $w_{i,j,k}$, in Eq. (S8) are chosen to resemble the physical process of Förster dipole-dipole energy transfer, the rate of which scales as d^{-6}, where d is the distance between NCs.[S15] The photon emission rate from a NC at a given position of (x,y,z), $S_{x,y,z}$, can be calculated from its occupancy, $N_{x,y,z}$, using

$$S(t) = R_X N_{x,y,z}(t)(1 + 4\delta_{N_{x,y,z}(t),2}). \tag{S10}$$

The predicted normalized luminescence efficiencies shown in Figs. 3**a**, 5**c**, and 6 are calculated by integrating $S(t)$ over time at different initial distributions of excitons.

Assuming that the exciton Bohr diameter in $CsPbBr_3$ NCs is around 7 nm,[S1] the critical density for exciton ionization (Mott density) is estimated to be $\sim 1.5 \times 10^{18}\,\mathrm{cm}^{-3}$,[S16] which corresponds to roughly 5 excitons per NC. To account for ultrafast free-carrier Auger processes and the breakdown of a well-defined exciton picture at higher occupancies, any NC initially populated with more than 5 excitons is, in our model, immediately reduced to 5 excitons at $t = 0$.

The biexciton lifetime, τ_{XX}, can be estimated through linear volume scaling, which for 15 nm cubic NCs yields 287 ps based on the studies presented in Ref. S17. However, other studies have shown that for NCs in the weak confinement regime ($V > 1000\,\mathrm{nm}^3$), τ_{XX} increases beyond the value predicted by linear volume scaling.[S18] Therefore, we choose a value of 350 ps for τ_{XX} in the simulations presented here.

The rate of biexcitonic emission (process d)) is obtained from straightforward combinatorial arguments, consistent with experimental observations.[S19] Radiative recombination from higher-order multiexcitons is excluded, in line with prior studies of densely-excited $CsPbBr_3$ NCs.[S14,S20,S21] The diffusion of excitons between NCs in the drop-cast sample (process e)) is assumed to be driven by Förster dipole-dipole energy transfer.[S22]

In all simulations, the number of excitons at $t = 0$ is 10^7, the absorption length is 10 NCs, and the beam width is varied from $10^2 - 10^4$ NCs. This replicates an initial peak density of excitons between 10^{-2} to 10^2 excitons per NC, which fits with the excitation density regime investigated in the $\mathcal{Z}$-scan luminescence measurements.

Table S1: The fitted exciton lifetimes and areas for the decay curves of all samples.

$n_{0,\max}$	τ_1 [ns]	A_1 [%]	τ_2 [ns]	A_2 [%]	τ_3 [ns]	A_3 [%]	τ_4 [ns]	A_4 [%]	$\boldsymbol{\tau_{\text{eff}}}$ **[ns]**
				Long ligands					
0.01	1.1(2.0)	7(11)	6.0(5.0)	45(33)	19(11)	45(33)	103(104)	4.6(5.2)	**6.4(2.0)**
0.1	1.6(2.8)	13(21)	7.9(10.9)	55(58)	22(33)	30(42)	161(453)	2.2(4.0)	**6.2(1.9)**
0.7	1.0(2.0)	10(18)	5.4(5.8)	52(39)	16(13)	36(44)	106(107)	2.4(3.9)	**4.6(1.6)**
12	0.4(0.7)	21(36)	2.3(3.0)	46(39)	8.8(13.7)	30(48)	56(141)	2.8(8.7)	**1.3(0.7)**
				Short ligands					
0.01	1.7(3.0)	8(13)	8.6(9.5)	44(42)	25(18)	44(47)	119(120)	3.9(5.9)	**8.8(2.4)**
0.1	1.6(2.9)	10(17)	9(10)	51(46)	25(27)	35(44)	118(119)	3.6(6.9)	**7.3(2.2)**
0.7	1.3(2.1)	14(23)	6.0(7.9)	50(41)	19(23)	32(43)	89(89)	4.0(8.1)	**4.7(1.5)**
12	0.3(0.6)	21(33)	2.0(2.6)	44(33)	9(20)	28(31)	40(91)	7(24)	**1.1(0.6)**
				High QY					
0.01	1.6(1.7)	7.6(8.9)	8.9(6.5)	29(10)	49(26)	35(10)	367(155)	29(7)	**10.8(3.2)**
0.06	1.3(1.7)	7.1(9.2)	7.9(6.2)	29(10)	45(24)	36(11)	331(133)	28(7)	**9.8(3.2)**
0.3	1.6(1.7)	14(16)	8.7(8.5)	35(14)	44(34)	32(16)	312(182)	20(8)	**7.3(2.1)**
12	0.3(0.5)	18(24)	2.1(3.1)	39(23)	13(19)	30(24)	94(95)	13(14)	**1.2(0.8)**
				Low QY					
0.01	2.2(2.4)	9(10)	13(12)	29(12)	67(46)	37(12)	469(370)	25(7)	**14.5(4.2)**
0.1	1.9(2.3)	8(10)	12(10)	29(11)	61(36)	38(11)	398(264)	25(8)	**13.3(3.9)**
1.7	1.2(1.3)	13(16)	6.5(6.6)	35(18)	32(34)	34(17)	142(143)	17(17)	**5.7(1.8)**
12	0.4(0.6)	20(25)	3.0(4.7)	38(24)	17(29)	29(24)	94(95)	12(18)	**1.6(0.9)**

S7 Time-resolved measurements and multiexponential fitting

To complement the time-integrated $\mathcal{Z}$-scan luminescence measurements, we also measure the decay kinetics from the samples at selected excitation densities using the same setup but switching the photodetector to a PicoQuant PMA07 Hybrid PMT operating in single-photon counting mode. The decay curves are fitted to a quadruple-exponential function convolved with the Gaussian impulse response function (IRF) of the hybrid PMT, analytically calculated in Ref. S23

$$f(t) = \sum_{i=1}^{4} \frac{A_i}{\tau_i} \exp\left(\frac{-t}{\tau_i}\right) * \mathrm{IRF}(t) \tag{S11}$$

$$= \sum_{i=1}^{4} \frac{A_i}{2\tau_i} \exp\left(\frac{-t}{\tau_i} + \frac{\sigma^2}{2\tau_i^2}\right) \left[1 - \mathrm{erf}\left(\frac{-\tau_i t + \sigma^2}{\sqrt{2}\sigma\tau_i}\right)\right], \tag{S12}$$

where A_i and τ_i are the areas and decay times of each decay component, respectively, and σ is the Gaussian width of the IRF measured separately to be around 30 ps, Multiexponential fits on the form of Eq. (S12) are shown for selected excitation densities in Figs. 3**a**, 3**b**, 4**a**, and 4**b**. The fits are obtained by using the `iminuit` package in python using a maximum-likelihood-based cost function. For completeness, the fit parameters for all fits made to the decay curves of the four samples are shown in Table S1.

From the fitted multiexponential decay components, an effective decay time is calculated as the amplitude-

weighted average decay time,

$$\tau_{\text{eff}} = \frac{\sum_{i=1}^{4} A_i}{\sum_{i=1}^{4} \frac{A_i}{\tau_i}}. \tag{S13}$$

Note that due to the highly correlated and nonlinear nature of the model, the full covariance matrix is needed for an accurate representation of the uncertainty associated with the estimate of the effective decay time. In practice, the uncertainty is calculated using

$$\sigma_{\tau_{\text{eff}}} = \sqrt{\nabla\tau_{\text{eff}}^{\mathrm{T}} \mathbf{C} \nabla\tau_{\text{eff}}}, \tag{S14}$$

where $\mathbf{C}$ is the numerically obtained covariance matrix and $\nabla^{\mathrm{T}} = \left(\frac{\partial}{\partial\tau_1}, \frac{\partial}{\partial A_1}, ..., \frac{\partial}{\partial\tau_4}, \frac{\partial}{\partial A_4}\right)$.

S8 Measurements using 511 keV excitation

For this study, 10 × 5 µL of $CsPbBr_3$ NCs dispersed in toluene (concentration of 15 mg/ml) were drop-cast on 3× 3 mm^2 BGO plate. Subsequently, polystyrene (PS) dissolved in toluene was drop-cast on top of the NC layer forming an optically transparent protective PS coating after drying. Then the sample was mounted to a NUV-MT SiPM (AFBR-S4N44P014M from Broadcom, 32 V breakdown voltage, 47 V bias voltage) using a Meltmount glue and wrapped with Teflon tape to improve light collection.

The experimental set-up used to measure the sample response under 511 keV γ-ray excitation is identical to the one described by Gundacker et al. in Ref. S24. The samples were placed on the opposite sides of a ^{22}Na source and measured in coincidence. For this study, the samples were measured in coincidence with a reference crystal (2 × 2 × 3 mm^3 LSO:Ce:Ca0.4%, 61 ps CTR FWHM).

The SiPM signal was read out by a high-frequency readout circuit, also described in Ref. S24, and finally digitized by a LeCroy DDA735Zi oscilloscope (3.5 GHz bandwidth, 20 Gs s^{-1} sample rate). More about the data processing can be found in Ref. S25.

The distinction of the pure $CsPbBr_3$ events and events with energy-sharing between the $CsPbBr_3$ and the BGO plate from pure BGO events was done using a charge-rise time correlation as shown in Fig. S1. In this depiction, the event selection can be done based on physical differences in scintillation mechanism and geometry of the materials. The NCs exhibit significantly faster rise and decay times compared to BGO, meanwhile the energy deposition in their thin layer is generally lower than in the scintillator plate leading to smaller charge of the events. This enables identification of two distinct regions, corresponding to the BGO events (depicted by the blue and green map on top right) and the $CsPbBr_3$/shared events (depicted by the red and yellow map on bottom left). This provides superior selection efficiency over the amplitude-charge correlation, in particular for the shared events where it is difficult to distinguish between the BGO and $CsPbBr_3$ events. The resulting selection of events from the charge-rise time correlation is highlighted in the amplitude-charge correlation in Fig. S2.

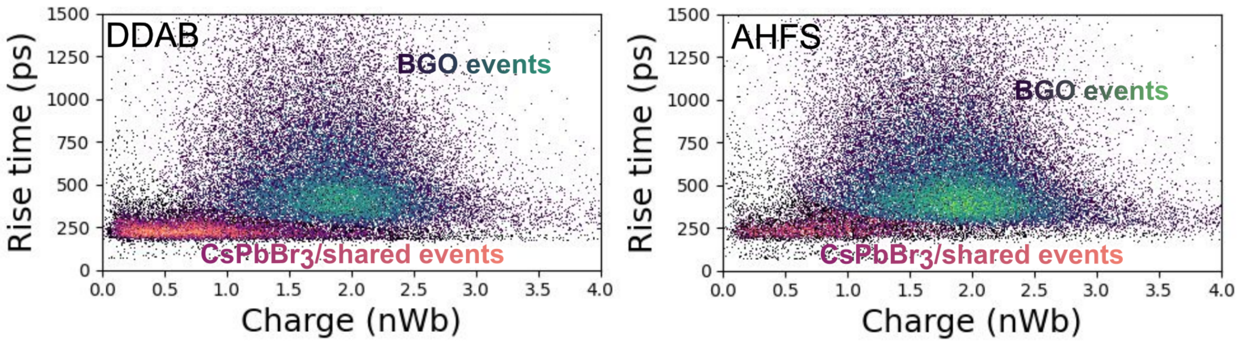

Figure S1: 2D rise time-charge histograms using layers of either DDAB- or AHFS-treated NCs on BGO scintillator. Selected regions corresponding to interactions purely in the BGO crystal and shared events are highlighted. Both histograms are normalized to the same total number of events.

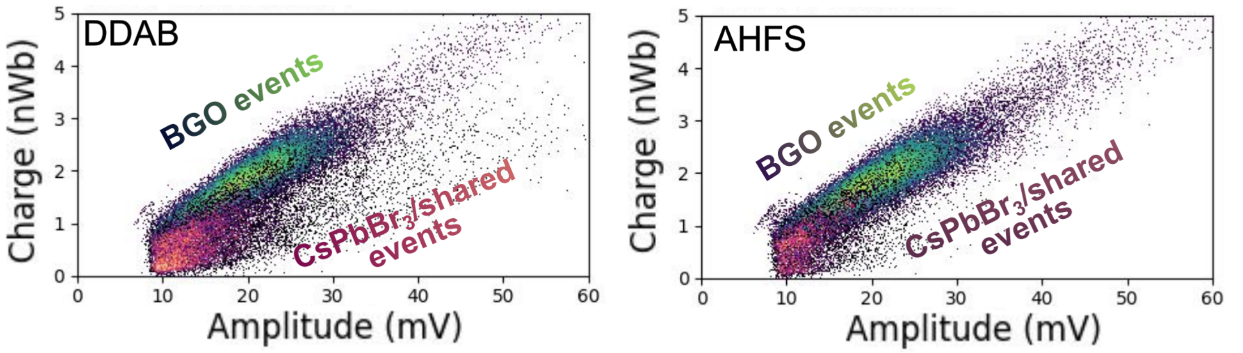

Figure S2: 2D charge-amplitude histograms using layers of either DDAB- or AHFS-treated NCs on BGO scintillator. Regions corresponding to interactions purely in the BGO crystal and shared events based on rise time selection presented in Figure S1 are highlighted. Both histograms are normalized to the same total number of events.

References

[S1] Protesescu, L.; Yakunin, S.; Bodnarchuk, M. I.; Krieg, F.; Caputo, R.; Hendon, C. H.; Yang, R. X.; Walsh, A.; Kovalenko, M. V. Nanocrystals of cesium lead halide perovskites ($CsPbX_3$, X = Cl, Br, and I): novel optoelectronic materials showing bright emission with wide color gamut. *Nano Lett.* **2015**, *15*, 3692–3696, PMID: 25633588.

[S2] Lu, C.; Wright, M.; Ma, X.; Li, H.; Itanze, D.; Carter, J. A.; Hewitt, C.; Donati, G.; Carroll, D.; Lundin, P.; Geyer, S. Cs oleate precursor preparation for lead halide perovskite nanocrystal synthesis: the influence of excess oleic acid on achieving solubility, conversion, and reproducibility. *Chem. Mater.* **2018**, *31*.

[S3] Děcká, K.; Král, J.; Hájek, F.; Průša, P.; Babin, V.; Mihóková, E.; Čuba, V. Scintillation response enhancement in nanocrystalline lead halide perovskite thin films on scintillating wafers. *Nanomaterials* **2021**, *12*, 14.

[S4] Král, J.; Děcká, K.; Liška, P.; Torres, S. R.; Valenta, J.; Babin, V.; Monzón, I. L.; Čuba, V.; Mihóková, E.; Auffray, E. Tailored thermally stable functionalization of $CsPbBr_3$ nanocrystals for polymer nanocomposite scintillator fabrication. *J. Mater. Chem. C* **2026**,

[S5] Kozma, I. Z.; Krok, P.; Riedle, E. Direct measurement of the group-velocity mismatch and derivation

of the refractive-index dispersion for a variety of solvents in the ultraviolet. *J. Opt. Soc. Am. B* **2005**, *22*, 1479–1485.

[S6] CAS source index (CASSI) search tool. `https://www.chemicalbook.com/ChemicalProductProperty_EN_CB9341362.htm`, Accessed: 15-12-2025.

[S7] Malitson, I. H. Interspecimen comparison of the refractive index of fused silica. *J. Opt. Soc. Am.* **1965**, *55*, 1205.

[S8] Achtstein, A. W.; Antanovich, A.; Prudnikau, A.; Scott, R.; Woggon, U.; Artemyev, M. Linear Absorption in CdSe Nanoplates: Thickness and Lateral Size Dependency of the Intrinsic Absorption. *J. Phys. Chem. C* **2015**, *119*, 20156–20161.

[S9] Lin, W.-H.; Pan, S.-C.; Hsu, J.-F.; Tseng, Z.-L.; Jyu, S.-S.; Lin, J.-H. Investigation of Two Photon Absorption of Ligand-Modified $CsPbBr_3$ Quantum Dots. *J. Phys. Chem. Lett.* **2022**, *13*, 11245–11252.

[S10] Chen, J.; Žídek, K.; Chábera, P.; Liu, D.; Cheng, P.; Nuuttila, L.; Al-Marri, M. J.; Lehtivuori, H.; Messing, M. E.; Han, K.; Zheng, K.; Pullerits, T. Size- and Wavelength-Dependent Two-Photon Absorption Cross-Section of CsPbBr3 Perovskite Quantum Dots. *J. Phys. Chem. Lett.* **2017**, *8*, 2316–2321.

[S11] Fu, Y.; Konda, S. R.; Ganeev, R. A.; Kim, V. V.; Boltaev, G. S.; Wang, R.; Yu, W.; Li, W. Outstanding nonlinear optical properties of all-inorganic perovskite CsPbX3 (X=Cl, Br, I) precursor solutions and polycrystalline films. *iScience* **2023**, *26*, 108514.

[S12] Imran, M.; Ijaz, P.; Goldoni, L.; Maggioni, D.; Petralanda, U.; Prato, M.; Almeida, G.; Infante, I.; Manna, L. Simultaneous Cationic and Anionic Ligand Exchange For Colloidally Stable CsPbBr₃ Nanocrystals. *ACS Energy Letters* **2019**, *4*, 819–824.

[S13] Jessen, S.; Di Giacomo, A.; Moreels, I.; Julsgaard, B.; Turtos, R. M. Nonlinear quenching of excitonic emission from nanoplatelet films at high excitation densities. *Sci. Rep.* **2025**, *15*, 23423.

[S14] Fratelli, A.; Zaffalon, M. L.; Mazzola, E.; Dirin, D. N.; Cherniukh, I.; Otero-Martínez, C.; Salomoni, M.; Carulli, F.; Rossi, F.; Meinardi, F.; Gironi, L.; Manna, L.; Kovalenko, M. V.; Brovelli, S. Size-dependent multiexciton dynamics governs scintillation from perovskite quantum dots. *Adv. Mater.* **2024**, *37*, 2413182.

[S15] Knox, R. S.; van Amerongen, H. Refractive index dependence of the Förster resonance excitation transfer rate. *J. Phys. Chem. B* **2002**, *106*, 5289–5293.

[S16] Mott, N. F. Metal-insulator transition. *Rev. Mod. Phys.* **1968**, *40*, 677–683.

[S17] Li, Y.; Ding, T.; Luo, X.; Chen, Z.; Liu, X.; Lu, X.; Wu, K. Biexciton auger recombination in monodispersed, quantum-confined $CsPbBr_3$ perovskite nanocrystals obeys universal volume-scaling. *Nano Res.* **2018**, *12*, 619–623.

[S18] Huang, P.; Sun, S.; Lei, H.; Zhang, Y.; Qin, H.; Zhong, H. Nonlocal interaction enhanced biexciton emission in large $CsPbBr_3$ nanocrystals. *eLight* **2023**, *3*, 10.

[S19] Li, B.; Huang, H.; Zhang, G.; Yang, C.; Guo, W.; Chen, R.; Qin, C.; Gao, Y.; Biju, V. P.; Rogach, A. L.; Xiao, L.; Jia, S. Excitons and biexciton dynamics in single $CsPbBr_3$ perovskite quantum dots. *J. Phys. Chem. Lett.* **2018**, *9*, 6934–6940.

[S20] Li, Q.; Yang, Y.; Que, W.; Lian, T. Size- and morphology-dependent auger recombination in $CsPbBr_3$ perovskite two-dimensional nanoplatelets and one-dimensional nanorods. *Nano Lett.* **2019**, *19*, 5620–5627.

[S21] Qin, C.; Jiang, Z.; Zhou, Z.; Liu, Y.; Jiang, Y. Excitation wavelength and intensity-dependent multiexciton dynamics in $CsPbBr_3$ nanocrystals. *Nanomaterials* **2021**, *11*, 463.

[S22] Penzo, E.; Loiudice, A.; Barnard, E. S.; Borys, N. J.; Jurow, M. J.; Lorenzon, M.; Rajzbaum, I.; Wong, E. K.; Liu, Y.; Schwartzberg, A. M.; Cabrini, S.; Whitelam, S.; Buonsanti, R.; Weber-Bargioni, A. Long-range exciton diffusion in two-dimensional assemblies of cesium lead bromide perovskite nanocrystals. *ACS Nano* **2020**, *14*, 6999–7007.

[S23] Gundacker, S.; Turtos, R.; Auffray, E.; Lecoq, P. Precise rise and decay time measurements of inorganic scintillators by means of X-ray and 511 keV excitation. *Nucl. Instrum. Methods Phys. Res. A* **2018**, *891*, 42–52.

[S24] Gundacker, S.; Turtos, R. M.; Auffray, E.; Paganoni, M.; Lecoq, P. High-frequency SiPM readout advances measured coincidence time resolution limits in TOF-PET. *Phys. Med. Biol.* **2019**, *64*, 055012.

[S25] Pagano, F.; Král, J.; Děcká, K.; Pizzichemi, M.; Mihóková, E.; Čuba, V.; Auffray, E. Nanocrystalline lead halide perovskites to boost time-of-flight performance of medical imaging detectors. *Adv. Mater. Interfaces* **2024**, 2300659.
